# Supplementary material for: NHR-14 loss of function couples intestinal iron uptake with innate immunity in C. elegans through PQM-1 signaling
Source: eLife. 2019 Sep 18;8:e44674. doi: 10.7554/eLife.44674 (PMC6777940; doi:10.7554/eLife.44674)
Supplement: Supplementary file 1. — C. elegans strains used in this study. This table lists worm strains generated in this study as well as previously referenced strains. [file elife-44674-supp1.docx]

| **Supplementary File 1 - Table S1: *C. elegans* strains used in this study** | | |
| --- | --- | --- |
| **Strain** | **Genotype** | **References** |
| N2 | Wild-type | (Brenner, 1974); PMID: 4366476 |
| CB4856 | Ancestral N2 Hawaiian Strain | WBPaper00005369  *C. elegans* Genetics Center |
| ZG31 | *hif-1(ia4)V* | (Jiang et al., 2001); PMID: 11427734 |
| *nhr-14(tm1473)* | *nhr-14(tm1473)X* | National Bioresource Project, Tokyo Womens Medical University |
| RB711 | *pqm-1(ok485)II* | WBPaper00041807 International *C. elegans* Gene Knockout Consortium |
| RB1074 | *smf-3(ok1035)IV* | WBPaper00041807 International *C. elegans* Gene Knockout Consortium |
| XA6904 | *pha-1 (e2123ts)III; qaEx6904 [Psmf-3(5’1500)*::*Δpes-10*::*GFP-his, pha-1+]* | (Romney et al., 2011); PMID: 18024960 |
| XA6905 | *pha-1(e2123ts) III; qaEx6905 [Psmf-3(5’ 250)*::*Δpes-10*::*GFP-his, pha-1+]* | (Romney et al., 2011); PMID: 18024960 |
| XA6906 | *hif-1(ia4)V; nhr-14(tm1473)X* | This paper |
| XA6907 | *smf-3(ok1035)IV; nhr-14(tm1473 )X* | This paper |
| XA6908 | *smf-3(ok1035)IV; hif-1(ia4)V; nhr-14(tm1473)X* | This paper |
| XA6909 | *ttTi5606; onc-119*(ed3)*III*; *hif-1(ia4)V* | This paper |
| XA6910 | *ttTi5606*; *unc-119* (ed3)*III*; *hif-1(ia4)V*; *nhr-14*(*tm1473)X* | This paper |
| XA6911 | *hif-1(ia4)V*, outcrossed to CB4856 | This paper |
| XA6912 | *nhr-14(tm1473)X,* backcrossed 6x times to wild-type N2 | This paper |
| XA6913 | *pha-1(e2123ts)III; qaEx6906 [Psmf-3(5’1500mDAE1)*::*Δpes-10*::*GFP-H2B, pha-1+]* | This paper |
| XA6914 | *pha-1(e2123ts) III; qaEx6907 [Psmf-3(5’1500mDAE2)*::*Δpes-10*::*GFP-H2B, pha-1+]* | This paper |
| XA6915 | *pha-1(e2123ts) III; qaEx6908 [Psmf-3(5’1500mDAE3)::Δpes-10::GFP-H2B, pha-1+]* | This paper |
| XA6916 | *hif-1(ia4)V; nhr-14(qa6909)X,* outcrossed to CB4856 | This paper |
| XA6917 | *hif-1(ia4)V; nhr-14(qa6910)X,* outcrossed to CB4856 | This paper |
| XA6918 | *ttTi5606; unc-119(ed3) III; qaEx6011[nhr-14[36443]*::*GFP*::*FLAG*::*unc-119-Nat]* | TransgeneOME and this paper |
| XA6919 | *ttTi5606*; *unc-119* *(ed3)III*; *hif-1(ia4)V*; *qaEx6011[nhr-14 [36443]*:: *GFP*::*FLAG*:: *unc-119-Nat]* | This study |
| XA6920 | *ttTi5606*; *unc-119* (ed3)*III*; *hif-1(ia4)V*; *nhr-14*(*tm1473)X; qaEx6011(nhr-14 [36443]::GFP*::*FLAG*::*unc-119-Nat)* | This study |
| OP201 | (*wgIs201* [*pqm-*1:: *TY1*::*EGFP*::*3xFLAG* (92C12)+*unc119*(+)]) | (Sarov et al., 2006); PMID: 16990816; TransgeneOME |
